# Recent Advances in Dual Temperature Responsive Block Copolymers and Their Potential as Biomedical Applications

**DOI:** 10.3390/polym8110380

**Published:** 2016-10-27

**Authors:** Yohei Kotsuchibashi, Mitsuhiro Ebara, Takao Aoyagi, Ravin Narain

**Affiliations:** 1 Department of Materials and Life Science, Shizuoka Institute of Science and Technology, 2200-2 Toyosawa, Fukuroi, Shizuoka 437-8555, Japan; kotsuchibashi.yohei@sist.ac.jp; 2 International Center for Materials Nanoarchitectonics (WPI-MANA), National Institute for Materials Science (NIMS), 1-1 Namiki, Tsukuba, Ibaraki 305-0044, Japan; EBARA.Mitsuhiro@nims.go.jp; 3 Graduate School of Industrial Science and Technology, Tokyo University of Science, 6-3-1 Niijuku, Katsushika, Tokyo 125-8585, Japan; 4 Department of Materials Engineering, Graduate School of Pure and Applied Sciences, University of Tsukuba, 1-1-1 Tennodai, Tsukuba, Ibaraki 305-8577, Japan; 5 College of Science and Technology, Nihon University, 1-8-14 Kanda Surugadai, Chiyoda-ku, Tokyo 101-8308, Japan; 6 Department of Chemical and Materials Engineering, University of Alberta, Edmonton, AB T6G 2G6, Canada

**Keywords:** dual thermoresponsive block copolymers, smart polymers, biomaterials

## Abstract

The development of stimuli responsive polymers has progressed significantly with novel preparation techniques, which has allowed access to new materials with unique properties. Dual thermoresponsive (double temperature responsive) block copolymers are particularly of interest as their properties can change depending on the lower critical solution temperature (LCST) or upper critical solution temperature (UCST) of each segment. For instance, these block copolymers can change from being hydrophilic, to amphiphilic or to hydrophobic simply by changing the solution temperature without any additional chemicals and the block copolymers can change from being fully solubilized to self-assembled structures to macroscopic aggregation/precipitation. Based on the unique solution properties, these dual thermo-responsive block copolymers are expected to be suitable for biomedical applications. This review is divided into three parts; LCST-LCST types of block copolymers, UCST-LCST types of block copolymers, and their potential as biomedical applications.

## 1. Introduction

Various types of synthetic polymers have been developed with unique physical, chemical, and biological properties for a range of applications. “Smart” polymers that can reversibly change their physicochemical properties by an external stimulus can detect even the slightest difference in physical or chemical properties such as temperature, pH, and molecular concentrations [1,2,3]. Thermo-responsive polymers belong to the class of the most studied smart polymers and they are classified as two types based on their thermo-responsive behavior i.e., lower critical solution temperature (LCST) and upper critical solution temperature (UCST) types [4]. The LCST types of thermo-responsive polymers can dissolve in aqueous solution at temperatures lower than the LCST and the polymers can become hydrophobic above the LCST, which is caused by accelerated polymeric intra- and inter-molecular hydrogen bonding/hydrophobic interactions resulting in disrupted hydrogen bonding interactions with surrounding water molecules. This reversible solubility change has been applied for a wide range of applications such as in the development of model proteins, triggers for self-assemblies, “on-off” switch of protein activities, cell sheet technologies, drug carriers, column chromatography, and sensors [5,6,7,8,9,10]. In contrast, UCST copolymers can dissolve in solvent above the UCST and are insoluble below the UCST. The phenomenon can occur via specific interactions such as hydrogen-bonding and electrostatic interaction. UCST copolymers are also applied in various fields such as assembled nanomaterials, sensors, and protein separations [4,11].

Various thermo-responsive polymers have been prepared either by designing new monomers to access the temperature responsive properties or by careful engineering of the polymeric structures. The relationship between the responsive temperature and types of polymers has been reported [4]. It is challenging to construct three-dimensional structures/functionalities similar to natural proteins and viruses using these thermo-responsive polymers. To achieve the bioinspired materials, understandably, the precise polymeric design is required with control over the molecular weights, LCST/UCST properties, compositions, and structures [12]. The development of controlled living radical polymerizations (CLRPs) such as nitroxide-mediated radical polymerization (NMP) [13], atom transfer radical polymerization (ATRP) [14,15], and reversible addition-fragmentation chain transfer (RAFT) polymerization [16,17] have facilitated access to functional polymers not only controlling the molecular weights and molecular weight distribution but also allowing the control of the polymer structures i.e., random, gradient, block, comb-like, branched, and cyclic polymers [1]. Moreover, with the introduction of click chemistry, more advanced polymer structures have been achieved with unique properties [18,19,20]. 

**Figure 1:**
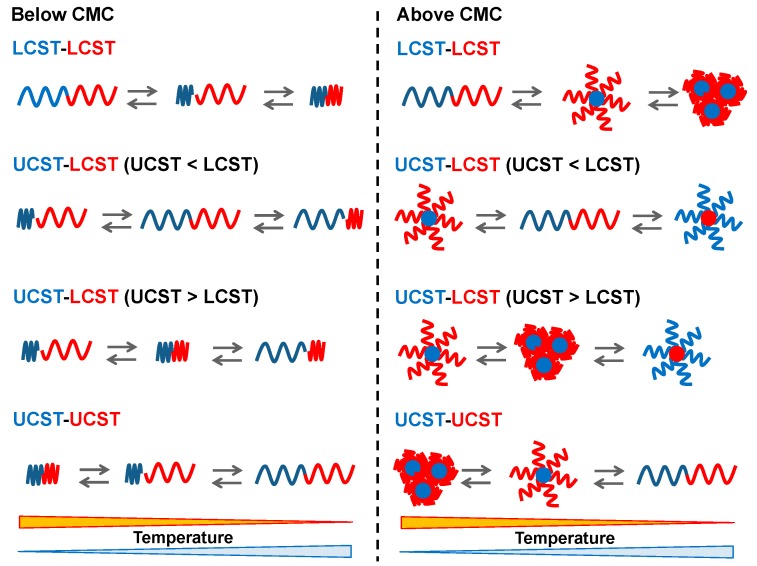
Conformation changes of dual thermoresponsive block copolymers at below or above thecritical micelle concentration (CMC).

**Figure 2:**
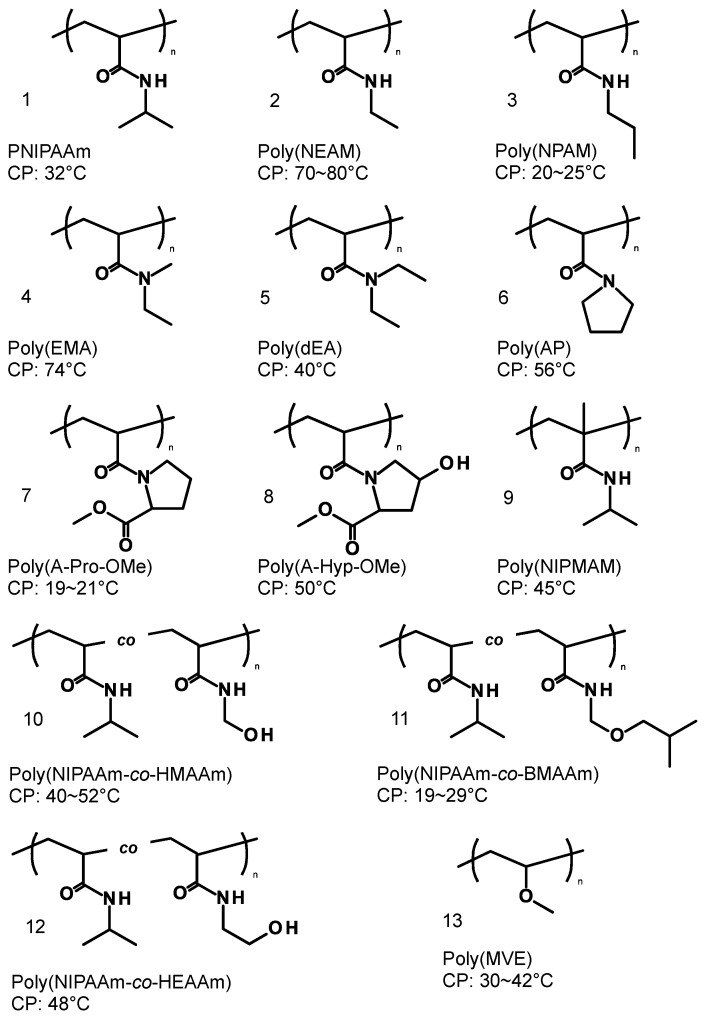

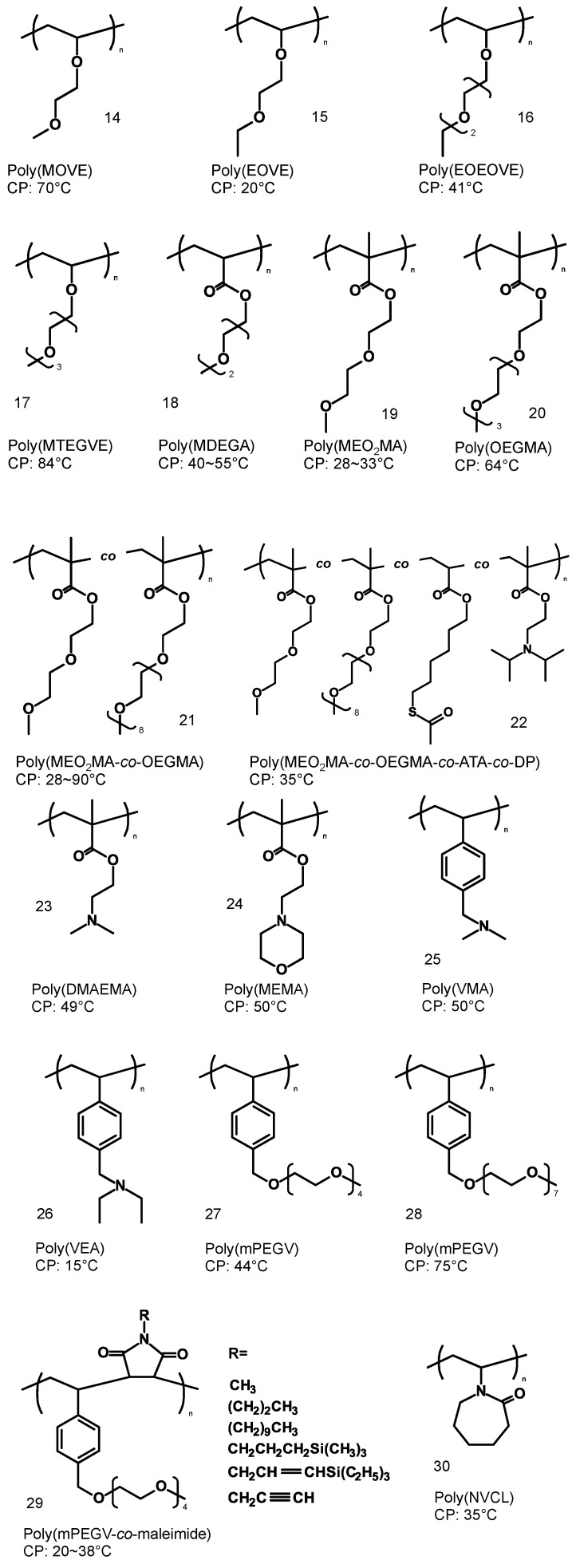

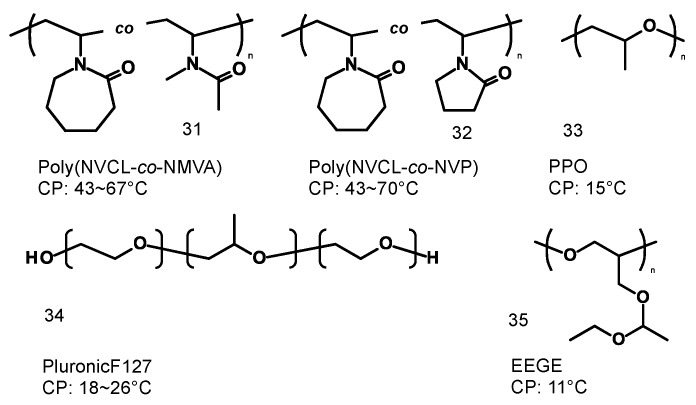
Structures of lower critical solution temperature (LCST) type of thermo-responsive copolymers for dual thermo-responsive block copolymers: **1**. NIPAAm: *N*-isopropylacrylamide; **2**. NEAM: *N*-ethylacrylamide; **3**. NPAM: *N*-*n*-propylacrylamide; **4**. EMA: *N*,*N*-ethylmethylacrylamide; **5**. dEA: *N*,*N*-diethylacrylamide; **6**. AP: *N*-acryloylpyrrolidine; **7**. A-Pro-OMe: *N*-acryloyl-l-proline methylester; **8**. A-Hyp-OMe: *N*-acryloyl-4-hydroxy-l-proline methylester; **9**. NIPMAM: *N*-isopropyl methacrylamide; **10**. HMAAm: *N*-hydroxymethyl acrylamide; **11**. BMAAm: *N*-(isobutoxymethyl) acrylamide; **12**. HEAAm: *N*-hydroxyethylacrylamide; **13**. MVE: methyl vinyl ether; **14**. MOVE: 2-methoxyethyl vinyl ether; **15**. EOVE: 2-ethoxyethyl vinyl ether; **16**. EOEOVE: 2-(2-ethoxy)ethoxyethyl vinyl ether; **17**. MTEGVE: methyltriethylene glycol vinyl ether; **18**. MDEGA: methoxydiethylene glycol acrylate; **19**. MEO_2_MA: 2-(2-methoxyethoxy) ethyl methacrylate; **20**–**21**. OEGMA: oligo(ethylethylene glycol)methyl ether methacrylate; **22**. ATA: 6-acethylthiohexylacrylate, DP: 2-(diisopropylamino)ethyl methacrylate; **23**. DMAEMA: 2-(dimethylamino)ethyl methacrylate; **24**. MEMA: 2-(*N*-morpholino)ethyl methacrylate; **25**. VMA: *N*-(4-vinylbenzyl)-*N*,*N*-dimethylamine; **26**. VEA: *N*-(4-vinylbenzyl)-*N*,*N*-diethylamine; **27**–**29**. mPEGV: poly(ethyleneglycol)methyl ether vinyl phenyl; **30**. NVCL: *N*-vinylcaprolactam; **31**. NMVA: *N*-methyl-*N*-vinylacetamide; **32**. NVP: *N*-vinylpyrrolidone; **33**. PPO: poly(propylene oxide); **34**. PluronicF127: poly(oxyethylene)-poly(oxypropylene)-poly(oxyethylene); **35**. EEGE: ethoxyethylglycidyl ether. CP: cloud point.

**Figure 3:**
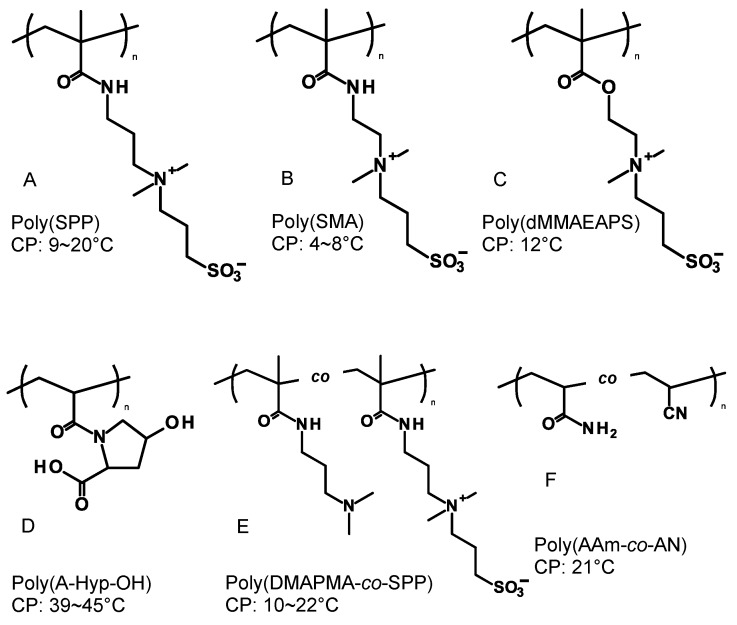

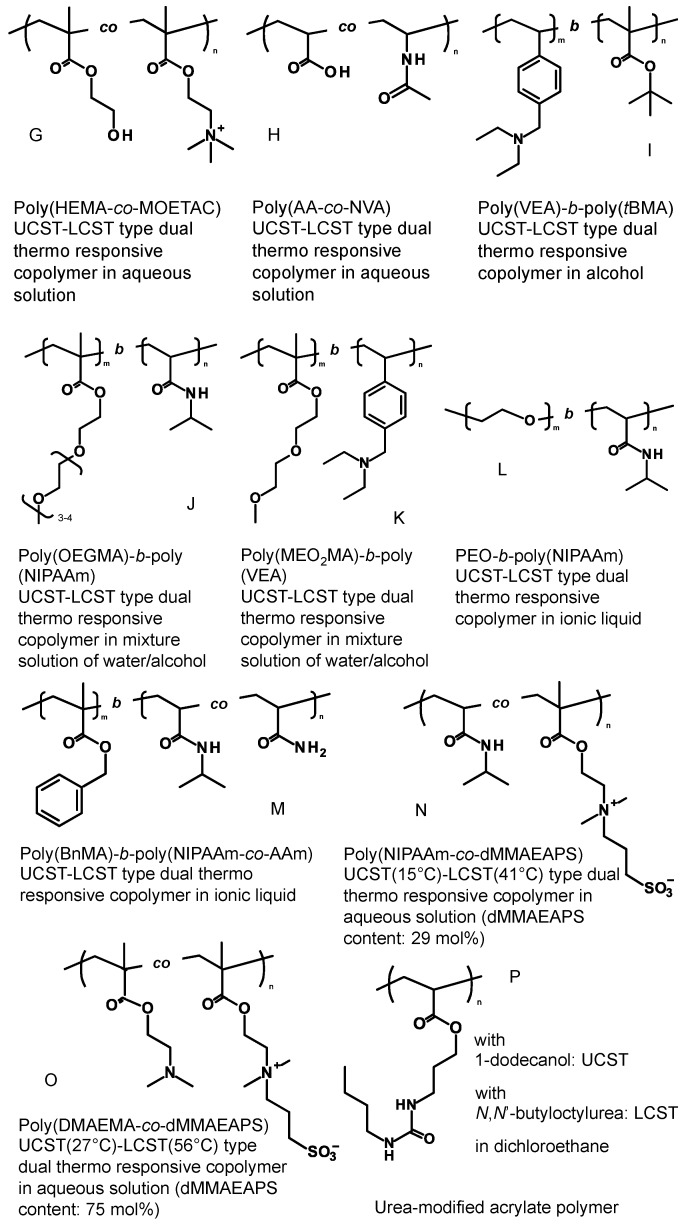
Structures of upper critical solution temperature (UCST) type of thermo-responsive copolymers for dual thermo-responsive block copolymers: **A**. SPP: 3-[*N*-(3-methacrylamidopropyl)-*N*,*N*-dimethyl]ammoniopropane sulfonate; **B**. SMA: sulfobetaine methacrylamide; **C**. dMMAEAPS: 3-dimethyl(methacryloyloxyethyl)ammonium propane sulfonate; **D**. A-Hyp-OH: *N*-acryloyl-4-trans-hydroxy-l-proline; **E**. DMAPMA: *N*-(3-(dimethylamino)propyl)methacrylamide; **F**. AAm: acrylamide, AN: acrylonitrile; **G**. HEMA: 2-hydroxyethylmethacrylate, MOETAC: [2-(methacryloyloxy)ethyl]trimethylammonium chloride; **H**. AA: acrylic acid, NVA: *N*-vinylacetamide; **I**. tBMA: tert-butyl methacrylate; **J**. OEGMA: oligo(ethylethylene glycol)methyl ether methacrylate, NIPAAm: *N*-isopropylacrylamide; **K**. MEO_2_MA: 2-(2-methoxyethoxy) ethyl methacrylate, VEA: *N*-(4-vinylbenzyl)-*N*,*N*-diethylamine; **L**. PEO: poly(ethylene oxide); **M**. BnMA: benzyl methacrylate; **N**. dMMAEAPS: 3-dimethyl(methacryloyloxyethyl)ammonium propane sulfonate; **O**. DMAEMA: 2-(dimethylamino)ethyl methacrylate; **P**. Urea-modified acrylate polymer. CP: cloud point.

The architectures of copolymers dictate the physicochemical properties even though they have the same chemical compositions. For example, poly(*N*-isopropyl acrylamide) (PNIPAAm) is one of the most studied LCST types of thermo-responsive polymers and the LCST was found to be different between linear- and cyclic-PNIPAAm [21,22]. Under the polymeric design, when two thermo-responsive segments are connected by a covalent bond at each end chain, the block copolymers are expected to possess a dual thermo-responsive property in the conformation changes. The dual thermo-responsive properties can be designed using the combinations of LCST and UCST types in each segment, i.e., LCST-LCST, UCST-LCST (UCST > LCST or UCST < LCST), and UCST-UCST (Figure 1). The temperature responsive behavior can be classified into liquid-solid phase transition and liquid-liquid phase separation due to the hydrophobicities [23]. Typical thermo-responsive homopolymers can broadly represent two property changes from hydrophilicity to hydrophobicity and hydrophobic interaction leads to aggregation/precipitation when the concentration is over the critical aggregation concentration (CAC). Dual thermo-responsive block copolymers can change the properties of each segment resulting in hydrophilic-hydrophilic, amphiphilic, and hydrophobic-hydrophobic structures depending on the solution temperature below the critical micelle concentration (CMC). At concentrations above the CMC, depending on the temperature, the dual thermo-responsive block copolymers can show assembled structural changes, which are dissolution, assembly, and aggregation/precipitation. In this way, these dual thermo-responsive copolymers can represent complex states/structures as compared to that of the thermo-responsive homopolymers. There are only a few reviews about dual thermo-responsive block copolymers and their phase transitions [24,25]. This review focuses on the current dual thermo-responsive block copolymers and their application in biomedical fields. The segment structures of block copolymers are shown in Figure 2 (for LCST-LCST) and Figure 3 (for UCST-LCST), and the combinations of dual thermo-responsive block copolymers are summarized in Table 1. The review is divided into three parts to cover LCST-LCST types of block copolymers, UCST-LCST types of block copolymers, and the potential as biomedical applications, particularly, drug carriers used in blood circulation. 

## 2. LCST-LCST Type Block Copolymers

**Figure 4:**
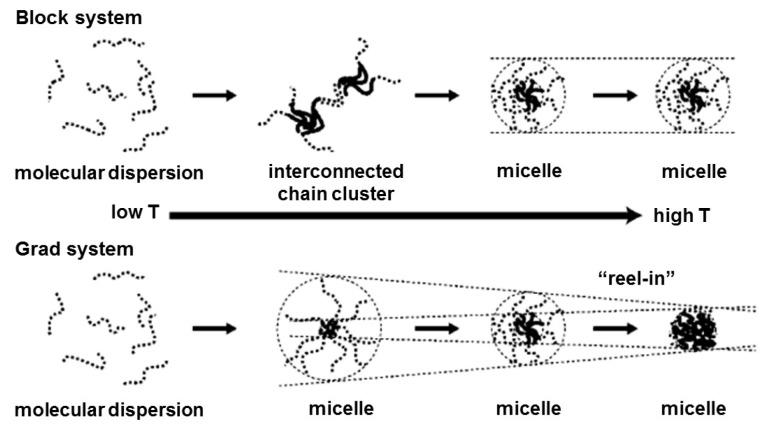
Micelle formation of block and grad copolymers in aqueous solutions. Reproduced with permission from Reference [29], Copyright 2006, American Chemical Society.

Amphiphilic block copolymers can form self-assembled core-shell nanoparticles in aqueous solution. Organic solvents are usually required to dissolve the amphiphilic block copolymers and the assembled nanostructures are formed once the organic solvents are removed by either evaporation or dialysis. For a more environmentally friendly approach, in recent years, much interest has been paid in the design of “stimuli-responsive” block copolymer systems that undergo a conformational change or phase transition in aqueous solution in response to an external stimulus (e.g., temperature, pH, light, and ionic strength) [1,2,3]. Among them, thermo-responsive polymers are the most studied stimuli-responsive polymers, which can show reversible hydration/dehydration at a temperature called the lower critical solution temperature (LCST) [4,6]. The nano-assemblies consisting of block copolymers having LCST type and hydrophobic segments can release loaded drugs from the core by the temperature resulting in the dehydration of the thermo-responsive segment. The block copolymers having a hydrophilic segment and a LCST type segment can encapsulate the drugs above the LCST. Therefore, a block copolymer having two LCSTs is expected to show both drug loading and release properties only by changing the temperature without any additional chemicals (with their multiple structural changes, i.e., dissolution, nanoassembly, and aggregation/precipitation). Moreover, these multiple formation changes lead to 3D nano-based constructions. Sugihara et al. controlled structural changes of the LCST-LCST oxyethylene-based block copolymers [26,27]. The block copolymers of poly(2-(2-ethoxy)ethoxyethyl vinyl ether)-*b*-poly(2-methoxyethyl vinyl ether) (poly(EOEOVE)-*b*-poly(MOVE)) changed their morphology depending on the temperature ranges, i.e., sol (<40 °C), transparent gel (42–55 °C), and clear liquid sol (57–63 °C). The gels were composed of the packed nanoparticles. Interestingly, the gel formation was strongly affected by the *M*_w_/*M*_n_. A block copolymer with a large *M*_w_/*M*_n_ (= 1.81) could not form a physical gelation [28]. Triblock copolymers having three different LCSTs were also prepared for a gel formation [30,31]. The morphology of a poly(EOVE_200_)-*b*-poly(MOVE_200_)-*b*-poly(EOEOVE_200_) was changed in solution (*T* < 20 °C), micellization (20 °C < *T* < 41 °C), physical crosslinking (41 °C < *T* < 61 °C), and precipitation (*T* > 64 °C) at 20 wt %. Different LCSTs usually mean different hydrophobicities. A triple thermo-responsive block copolymer of poly(*N*-*n*-propylacrylamide (nPA))-*b*-PNIPAAm-*b*-poly(*N*,*N*-ethylmethylacrylamide (EMA)) (LCSTs: 31.5, 46.8, and 57.6 °C) was measured on thermal transitions using differential scanning calorimetry (DSC) [32,33]. The homopolymers of the poly(nPA) and PNIPAAm showed single peaks in the DSC curves due to each dehydration. Interestingly, a single peak at 24 °C was observed in the triblock copolymer. The continued poly(nPA) and PNIPAAm segments led to the single peak by their continuous dehydration and no enthalpy change was observed at the poly(EMA) segment due to its weak hydrophobicity. When the chain length of one segment is too short as compared to the other neighboring segment, the thermo-responsive behavior can be affected [35]. For instance, the LCST of PNIPAAm with hydrophobic pyrene as the end group was dramatically decreased (from 29.3 to ~21.7 °C) with decreasing molecular weight (5000–3000 g/mol) [83]. Chain-ends of the LCST-LCST type block copolymers were capped with trimethylsilyl (TMS) groups for tracing the temperature responsive behavior [38,39]. The poly(*N*-*n*-propylacrylamide (NPAM))-*b*-poly(*N*-ethylacrylamide (NEAM)) showed each LCST around 20 and 70 °C, respectively. The ^1^H nuclear magnetic resonance (NMR) peak of the TMS (0.16 ppm) located at the poly(NPAM) side disappeared due to dehydration with a temperature increase to 32 °C. The combination of ^1^H NMR and TMS end groups could provide additional information not only about the primary structure of the copolymers including unit numbers, molar mass, and the end functionalities but also the self-assembly process. Okabe et al. compared a temperature responsive behavior between a block copolymer and a gradient copolymer by DLS and SANS measurements [29]. The copolymers were LCST-LCST poly(EOVE)-*b*-poly(MOVE) (poly(EOVE) segment: LCST around 20 °C, poly(MOVE) segment: LCST around 60 °C, and poly(EOVE-*grad*-MOVE). The MOVE composition was gradually increased in the poly(EOVE-*grad*-MOVE) structure. The poly(EOVE)-*b*-poly(MOVE) clearly showed a dual transmittance decrease with increasing solution temperature. In the poly(EOVE-*grad*-MOVE), the transmittance was decreased gradually from 23 °C. The diameters of gradient copolymers were decreased with increasing solution temperature. On the other hand, the block copolymers kept their diameters. The small size in the gradient copolymer particles was explained as the “reel-in” effect (Figure 4).

**Figure 5:**
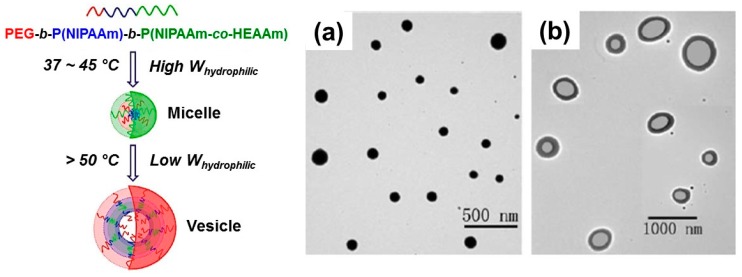
Transmission electron microscope (TEM) images of (**a**) micelles and (**b**) vesicles at 45 and 60 °C. Reproduced with permission from Reference [40], Copyright 2012, Royal Society of Chemistry.

**Figure 6:**
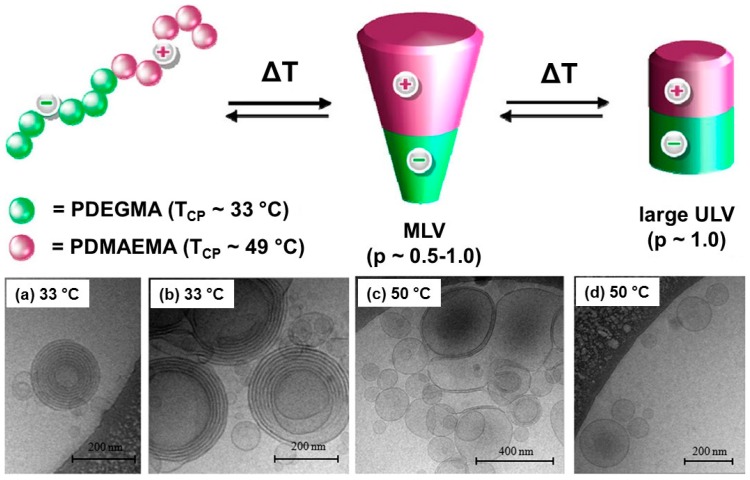
Cryo-TEM images of (**a**,**b**) multilamellar vesicles (MLV) and (**c**,**d**) unilamellar vesicles (ULV) at 33 and 50 °C. Reproduced with permission from Reference [41], Copyright 2012, American Chemical Society.

The hydrophilic/hydrophobic balance strongly affects the self-assembled nanostructures. In amphiphilic block copolymers, the assembled structures were turned from micelle to vesicle with increasing hydrophobic segment ratio because of the thermodynamic stability [84]. Wei et al. prepared an LCST-LCST type triblock copolymer poly(ethylene glycol)(PEG)_45_-*b*-PNIPAAm_380_-*b*-poly(NIPAAm_423_-*co*-*N*-hydroxyethyl acrylamide (HEAAm)_42_) [40]. The hydrophobic region in the triblock copolymer was controlled by the continuous dehydration of the PNIPAAm and poly(NIPAAm-*co*-HEAAm) segments that showed LCST at 37 and 48 °C, respectively. The assembled structures were converted between micelle (37–45 °C, around 260 nm) and vesicle (over 50 °C, ~420 nm) (Figure 5). Pietsch et al. prepared charge-controlled LCST-LCST block copolymers consisting of poly(2-(dimethylamino)ethyl methacrylate (DMAEMA)) and poly(di(ethyleneglycol)methyl ether methacrylate (DEGMA)) segments [41]. The poly(DMAEMA) (cloud point ~49 °C) and poly(DEGMA) (cloud point ~33 °C) segments were charged with positive and negative charges, respectively. The charge density was changed resulting in the temperature responsive dehydration, which led to unique assembled structures of multi-lamellar vesicular (MLV) aggregates and unilamellar vesicle (ULV) structures at 33 and 55 °C (Figure 6).

**Figure 7:**
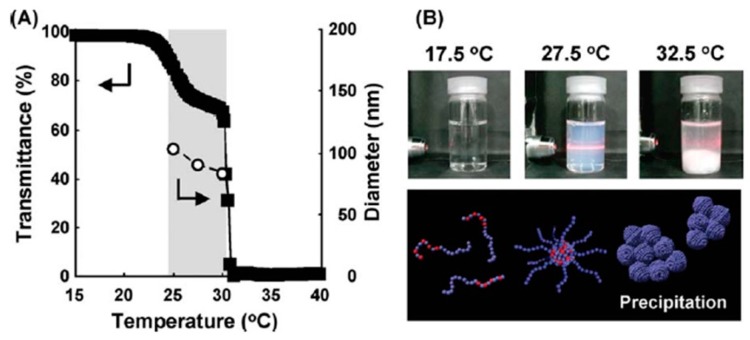
(**A**) Transmittance and diameter changes of P(NIPAAm)-*b*-P(NIPAAm-*co*-BMAAm)s (0.5 wt % aqueous solution) as a function of temperature (closed square: transmittance, open circle: diameter); (**B**) Photographs of 0.5 wt % aqueous solution of the bock copolymer at different temperatures. Reproduced with permission from Reference [51], Copyright 2010, John Wiley and Sons.

In this way, dual temperature responsive block copolymers were prepared, and the complex structures/functionalities were designed after the development of living radical polymerization, click chemistry, and their combinations [34,42,43,44,45,46,47,48,49,50,85,86]. We prepared LCST-LCST block copolymers consisting of PNIPAAm and poly(NIPAAm-*co*-*N*-hydroxymethyl acrylamide (HMAAm)) segments by one-pot ATRP [35,36]. The LCSTs of the poly(NIPAAm-*co*-HMAAm) segments were adjusted by the hydrophilic HMAAm contents, and the two step thermo-responsive properties were observed by ^1^H NMR spectra. Nanoassemblies have to be used above LCST of the PNIPAAm segment to maintain the assembled structures. To overcome this problem, hydrophobic *N*-(isobutoxymethyl) acrylamide (BMAAm) was copolymerized in the LCST-LCST block copolymer of poly(NIPAAm-*co*-BMAAm)-*b*-PNIPAAm [51,87]. At 27.5 °C, the block copolymer formed nanoassemblies (around 80 nm), which were observed to have a structure consisting of a poly(NIPAAm-*co*-BMAAm) core and a PNIPAAm shell by ^1^H NMR. Moreover, aggregation/precipitation was observed at 32.5 °C due to the dehydration of the PNIPAAm shell (Figure 7). 

Unique synthesis methods for dual thermos-responsive copolymers were also reported. Weiss et al. utilized a reaction ratio between monomers for the one-step preparation of LCST-LCST block copolymers [52]. It was reported that a block copolymer could be prepared under a mixed monomer condition of maleic anhydrate with superabundant styrene [88,89,90]. The compound 4-vinylbenzyl methoxytetrakis(oxyethylene) ether was mixed with various *N*-substituted maleimides and the block copolymers were successfully polymerized. Kermagoret et al. prepared *N*-vinylcaprolactam-based dual thermo-responsive block copolymers by a one-step synthesis method using cobalt-mediated radical polymerization [53,54]. Using a combination of living cationic and reversible addition-fragmentation chain transfer (RAFT) polymerizations, Sugihara et al. prepared LCST-LCST block copolymers of poly(MOVE)-*b*-PNIPAAm [55,56]. The conversion from living cation polymerization to RAFT polymerization was achieved by using carboxylic RAFT agents. Li et al. prepared a hetero-arm star polymer P(St)-PNIPAAm-P(DMAEMA) (PNIPAAm segment: LCST 32 °C, P(DEAEMA) segment: LCST 40–50 °C) [57]. The P(St)-*b*-PNIPAAm that has an alkyne group at the junction was connected with poly(DMAEMA) having an azide group at the end of the polymer chain by click chemistry. The assemblies were composed of both PNIPAAm and P(DMAEMA) segments on the surface and the diameters decreased from 77 to 40 nm with two steps. Xu et al. prepared the LCST-LCST block copolymers of PNIPAAm-*b*-P(DMA) (PNIPAAm segment: LCST 32 °C, P(DMA) segment: LCST 40–50 °C) from a hyperbranched polyester (Bolton H40) [58]. With increasing solution temperature, two stages of thermally induced collapse were observed by LLS, micro-DSC, and fluorescent measurement. Dendric H40 having PNIPAAm homopolymers was also prepared by the same researchers [91]. Interestingly, the H40-PNIPAAm also showed dual temperature size decrease. This behavior was due to the pseudo-high concentration of the inner layer of the PNIPAAm as compared to that of the outer layer. The difference in polymer density led to the two step temperature responsive properties. The same phenomena were observed for other nanoparticles having temperature responsive homopolymers [92,93,94].

## 3. UCST-LCST Type Block Copolymers

LCST-LCST (LCST 1 >LCST 2) types of dual thermo-responsive block copolymers can change their structures depending on the solution temperature, i.e., *T* < LCST 1: dissolution, LCST 1 < *T* < LCST 2: core-shell micelle, and *T* > LCST 2: aggregation/precipitation. Each segment always changes its properties from hydrophilic to hydrophobic. Therefore, the first LCST segment can be a core of the micelle but cannot be a shell at any temperature in water. The fixed position of the micelle core/shell occurs in all amphiphilic block copolymers. The inversion of the micelle core/shell, therefore, is a challenge in a self-assembly system. To achieve core/shell exchange, the difference of solubility at each segment in a block copolymer was focused on. Oranli et al. prepared a styrene-butadiene block copolymer and the relationship between core and shell of the micelle was reversed depending on the organic solvent (poly(St)) core in *n*-alkanes, poly(butadiene) core in *N*,*N*-dimethylformamide (DMF), *N*,*N*-dimethylacetamide (DMA), and methylethylketone (MEK) [95]. Bütün et al. achieved the micelle inversion in aqueous solution using pH and the salt responsive block copolymer of poly(2-(*N*-morpholino)ethyl methacrylate (MEMA))-*b*-poly(2-(diethylamino)ethyl methacrylate (DEAEMA) [96]. The micelle core was exchanged at room temperature by adjusting the solution pH and the electrolyte concentrations. 

These inversion systems of micelles are required to add organic solvents and/or to change the solution pH. To achieve the inversion system of micelle without any adding materials, polymers having an upper critical solution temperature (UCST) have been focused on. UCST type thermo-responsive polymers can dissolve in solvents at temperatures above the UCST and become insoluble below the UCST. This mechanism is explained via specific interactions such as hydrogen-bonding and electrostatic interaction [11]. Poly(sulfobetaine methacrylate (SBMA)) is one of the most studied UCST type temperature responsive polymers, which has a zwitterionic betaine group (CH_2_CH_2_N^+^(CH_3_)_2_CH_2_CH_2_CH_2_SO_3_^−^) in the structure [97]. The UCST of poly(SBMA) is triggered by the charge-charge and dipole-dipole interactions of the betaine groups. 

Block copolymers having both UCST and LCST (i.e., UCST-LCST block copolymers) are expected to form switchable core-shell micelle structures depending on the solution temperature without any additives. The UCST-LCST block copolymer was reported first by Arotçaréna et al. [63]. The block copolymers of PNIPAAm-*b*-poly(3-[*N*-(3-methacrylamidopropyl)-*N*,*N*-dimethyl]ammoniopropane sulfonate (SPP)) were synthesized at different chain lengths of the poly(SPP) segments and showed both UCST (poly(SPP) segment: 9–20 °C) and LCST (PNIPAAm segment: 33–34 °C) in aqueous solution, respectively. The relationship between UCST and LCST was always UCST < LCST. At below the UCST, therefore, hydrophobic the poly(SPP) segment was located in the micelle core surrounded with hydrophilic PNIPAAm shell. At temperatures over the LCST, the core and shell were exchanged to become poly(SPP) shell and PNIPAAm core due to the change in the solubilities of each segment. The block copolymers were completely dissolved in water at temperature between the UCST and LCST. These solution behaviors were measured using turbidity, ^1^H NMR, viscosity, and fluorescent intensity. 

Both UCST and LCST are strongly affected not only by the polymeric structures but also the solution conditions. The UCSTs of the poly(betaine) family, for example, are decreased in KCl solution due to the water-soluble improvement of the whole poly(betaine) resulting in interaction between negative charged chloride ions (Cl^−^) and quaternary ammonium groups in the polymers [98]. The LCST of PNIPAAm is decreased by adding NaCl, which is well known as salting-out effect. The salting-effect to the LCST has been reported using several kinds of salts, i.e., Hofmeister series [99,100,101]. Virtanen et al. investigated the NaCl salting-effect of UCST-LCST block copolymer of PNIPAAm-*b*-poly(SPP) (poly(SPP) segment: UCTS 9 °C, 9700 g/mol, and PNIPAAm segment: LCST 32 °C, 10,800 g/mol) [64]. Both the UCST and LCST were affected due to the NaCl concentrations. By increasing the NaCl concentration, the LCST was decreased by the salting-out effect, and the UCST of the poly(SPP) segment finally disappeared due to its hydrophilicity. Weaver et al. prepared poly(2-(dimethylamino)ethyl methacrylate)-*b*-poly(2-(*N*-morpholino)ethyl methacrylate)(poly(DMA)-*b*-poly(MEMA)) as a precursor for UCST-LCST block copolymer [65]. Continuously, the poly(DMA) segment was modified by 1,3-propane sultone to show the UCST and the precursors successfully converted to UCST-LCST type of poly(SBMA)-*b*-poly(MEMA) block copolymer (poly(SBMA) segment: UCST 20 °C, and poly(MEMA) segment: LCST 50 °C). The diameters of the switchable micelles were 50 nm (*T* < UCST, PSBMA core) and 42 nm (*T* > LCST, PMEMA core), respectively. By infrared spectroscopy (IR), Maeda et al. investigated the formation of a UCST-LCST block copolymer of poly(3-dimethyl(methacryloyloxyethyl)ammonium propane sulfonate)-*b*-poly(*N*,*N*-diethylacrylamide)(poly(dMMAEAPS)-*b*-poly(dEA)) (poly(dMMAEAPS) segment: UCST around 12 °C, and poly(dEA) segment: LCST around 40 °C) [66]. The IR peaks on the UCST were traced from ν(SO_3_^−^) and ν(C=O)_ester_ in the poly(dMMAEAPS) segment and a peak of amide-I in poly(dEA) segment was used for the LCST. According to an equation (ΔΔA = ΔA_T-T0_(ν_1_) − ΔA_T-T0_(ν_2_): ΔA_T-T0_(ν_1_) and ΔA_T-T0_(ν_2_) denoted absorbance values measured by IR), the ΔΔA values on ν(SO_3_^−^), ν(C=O)_ester_, and amide-I were increased at each temperature, which agreed well with the UCST and LCST measured using a transmittance change. Since the appearance of first UCST-LCST block copolymer, the polymeric structures/functionalities have been complicated and new types of UCST-LCST block copolymers have been designed not only for the basic polymer chemistry but also for different types of applications [73,74,102,103]. Mori et al. prepared the proline-based LCST-UCST block copolymers of poly(*N*-acryloyl-l-proline methyl ester)-*b*-poly(*N*-acryloyl-4-*trans*-hydroxy-l-proline) (poly(A-Hyp-OMe)-*b*-poly(A-Hyp-OH)) (poly(A-Hyp-OMe) segment: LCST 19–21 °C, and poly(A-Hyp-OH) segment: UCST 39–45 °C). The LCST-UCST type of block copolymers was easily converted to LCST-LCST block copolymers by the methylation reaction [59]. Tian et al. controlled each UCST and LCST in a block copolymer by adjusting the compositions (poly(2-(2-methoxyethoxy)ethyl methacrylate-*co*-OEGMA)-*b*-poly(*N*-(3-(dimethylamino) propyl) methacrylamide) (poly(MEO_2_MA-*co*-OEGMA)-*b*-poly(DMAPMA)) [67]. The UCSTs and LCSTs were linearly increased on increasing the degree of quaternization of the DMAPMA segment (quaternization: 35%–70%, UCST 10–22 °C) and the contents of OEGMA (OEGMA: 0%–30%, LCST 28–60 °C), respectively. 

UCSTs of betaine-based copolymers are strongly affected by the addition of salts due to the charged structures. To overcome this limitation, a UCST copolymer series that is of hydrogen bonding-based uncharged copolymers has been reported [104,105,106]. The mechanism of UCST comes from the balance of hydrogen-donor (such as NH, NHs) and hydrogen-acceptor (such as O), which is dependent on the polymeric structures (Figure 3). Seuring and Agarwal reported an uncharged UCST copolymer consisting of commercially available acrylamide (AAm) and acrylonitrile (AN) monomers [107]. Zhang et al. combined an UCST poly(AAm-*co*-AN) segment with a hydrophilic (or hydrophobic) segment, and the block copolymers formed poly(AAm-*co*-AN) core (or shell) micelles in a physiological medium. Moreover, the poly(AAm-*co*-AN) segment was also combined with PDMAEMA segment and showed a solution behavior of the UCST-LCST type [68]. Very recently, Käfer et al. prepared a UCST-LCST type of PEG-*b*-poly(AAm-*co*-AN) block copolymer by free radical polymerization using a PEG macro-azo-initiator [69]. Conventional free radical polymerization has a big advantage for mass production.

**Figure 8:**
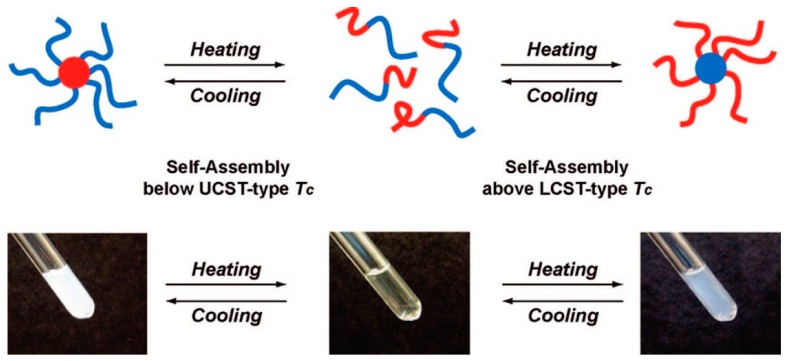
Concept of a dual thermo-responsive self-assembly in a single solvent. The red and blue blocks show UCST-type and LCST-type phase behavior, respectively. Reproduced with permission from Reference [78], Copyright 2009, American Chemical Society.

**Figure 9:**
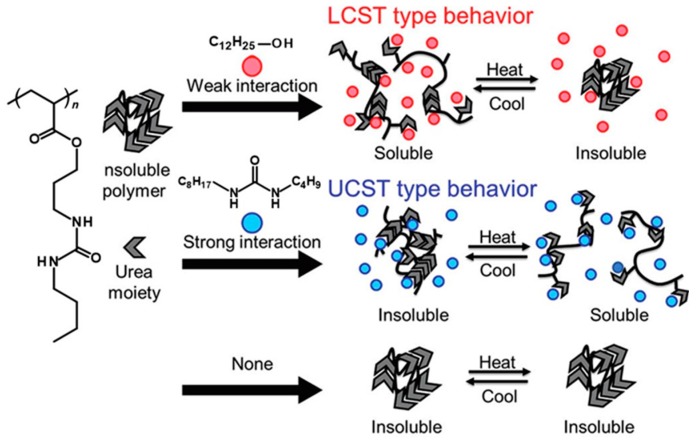
Themo-responsive polymers with LCST-type and UCST-type behavior controlled by the additives. Reproduced with permission from Reference [80], Copyright 2012, American Chemical Society.

UCST-LCST block copolymers show temperature responsive properties not only in aqueous solution but also in other solvents such as organic solvents and ionic liquids, which are applied in catalysis, batteries, and devices as sustainable materials [75,76,77,78]. Roth et al. prepared poly(OEGMA)-*b*-PNIPAAm and poly(OEGMA)-*b*-poly(*N*,*N*-diethylacrylamide (DEAM)) block copolymers and showed a system both of UCST and LCST in a mixture water/alcohol solution [76]. The poly(OEGMA) segment showed UCST in alcohol and PNIPAAm/poly(DEAM) segments showed LCSTs in water, respectively. Su et al. prepared a block copolymer poly(MEO_2_MA)-*b*-poly(*N*-(4-vinylbenzyl)-*N*,*N*-diethylamine (VEA)) where each segment can switch temperature responsive behavior to UCST or LCST depending on the solvents such as water, alcohol, and the mixture of solvents [77]. Poly(MEO_2_MA) segment, for example, showed a LCST in water and a UCST in isopropanol, respectively. On the other hand, the poly(VEA) segment had a LCST in mixture water/isopropanol and a UCST in isopropanol. The temperature-dependent morphology (dissolution, nanoassemblies, and aggregation/precipitation) of the block copolymer was investigated at different mixture ratios of water/isopropanol. Ueki et al. focused on ionic liquids as an activity environment for UCST-LCST block copolymers (Figure 8) [78]. Ionic liquids have had much attention paid for their unique abilities such as low volatility, non-flammability, high ion conductivity, and temperature/chemical stability [108,109]. The block copolymer poly(benzyl methacrylate (BnMA))-*b*-PNIPAAm was dissolved in the ionic liquid of 1-ethyl-3-methylimidazolium bis(trifluoromethane sulfonyl)imide ([C_2_min][NTf_2_]), 1-butyl-3-methylimidazolium hexafluorophosphate ([C_4_min]PF_6_), and their mixture. In the case of a mixture of the solvents ([C_2_min][NTf_2_]/[C_4_min]PF_6_ = 1:1), the UCST (PNIPAAm segment) and LCST (poly(BnMA) segment) were 30 and 130 °C, respectively. Moreover, the UCST and the assembly diameter were controlled via copolymerizing the PNIPAAm segment with an AAm monomer. The UCST-LCST temperature response in ionic liquids can construct a separation system without evaporation of the solvent. Moreover the nanoassemblies may travel back and forth between ionic liquid and water layers by controlling the solution temperature. Lee et al. prepared a UCST-LCST block copolymer of PEO-*b*-PNIPAAm and investigated its thermal morphology in ionic liquids of 1-ethyl-3-methylimidazolium tetrafluoroborate ([EMIM][BF4]), 1-butyl-3-methylimidazolium tetrafluoroborate ([BMIM][BF4]), and their blends (PEO segment: LCST, and PNIPAAm segment: UCST) [79]. Both LCST and UCST were shifted depending on the mixture ratios of ionic liquids and they found a mixture condition for exchange of LCST and UCST (i.e., LCST > UCST or LCST < UCST). The supra-molecular compounds have also been focused on as a new dual thermo-responsive system. Das et al. succeeded in showing both UCST and LCST in a supra-molecular compound in an organic solvent using a combination of hydrogen-bonding and π–π interaction [110]. Naphthalene diimide having hydrazide and hydroxy groups formed vesicle, micelles, and aggregation depending on the solution temperature. Moreover, the formation can be changed upon addition of pyridine-based compounds for the donor-acceptor complex. Amemori et al. prepared a urea-modified acrylate copolymer [80]. The copolymer could switch the LCST or UCST types of behavior in 1,2-dichloroethane (DCE) by the addition of small molecules (Figure 9). The LCST type behavior was achieved by adding alcohols due to the weak interaction with the urea moiety. On the other hand, molecules such as aliphatic carboxylic acids, dialkylureas, and tetrahexylammonium bromide acquired UCST type behavior because of their strong interaction. Interestingly, the copolymer showed both UCST and LCST in the presence of 1-dodecanol (weak interaction) and *N*,*N*’-butyloctylurea (strong interaction).

## 4. Potential of Dual Thermo-Responsive Block Copolymers as Biomedical Applications

Dual thermo-responsive block copolymers have been investigated as advanced biomaterials due to their interesting properties. The dehydration behavior of PNIPAAm, for example, has been used as a signal of surface plasmon resonance (SPR) for a biosensing application, which can be applied in a rapid/sensitive diagnosis system [111,112]. Jochum et al. prepared biotin modified LCST-LCST block copolymers of poly(oligo(ethylene glycol) monomethyl ether methacrylate (OEGMA))-*b*-poly(*N*-isopropyl methacrylamide (NIPMAM))-biotin [60]. Using a streptavidin immobilized surface (thickness: 0.00 ± 0.09 nm), the adsorption of the P(OEGMA)-*b*-P(NIPMAM)-biotin was measured by SPR at different temperatures. The adsorption test was performed at 1 mg/mL polymer concentration in PBS for 3 h. At 25 °C, the film thickness was increased to 0.22 nm due to biotin-avidin interaction. The film thickness was 0.01 ± 0.07 nm at 50 °C at which the biotin was encapsulated in the micelle core. These thermo-responsive devices have the potential to drive diagnosis by body temperature, which means an energy free diagnosis. Dual (or multi) thermo-responsive properties can express a complex signal and it will work to avoid an error in the diagnosis. It is also useful that polymers can be easily combined with inorganic particles. Paulus et al. polymerized PNIPAAm-based tri-block copolymers from iron particles for a biological separation system [113]. Dong et al. prepared a copolymer of P(SBMA-*co*-DMAEMA) from silica nanoparticles (SiNPs) by surface initiated ATRP [81]. SiNPs have huge potential in a wide range of fields such as drug carriers, imaging, sensors, and catalysis due to the ease of controlling the size, shape, surface modification, and their biocompatibility [114,115,116].

For clinical applications, blood compatibility, i.e., preventing adsorptions of non-specific proteins, such as fibrinogen and clotting enzymes, is a necessary criterion. These plasma proteins play an important role in polymer-induced clotting. Non-fouling polymers are required to possess properties such as hydrophilicity, neutral charge, and be hydrogen-bond acceptors rather than donors; PEG is one of the most studied non-fouling polymers [117].

**Figure 10:**
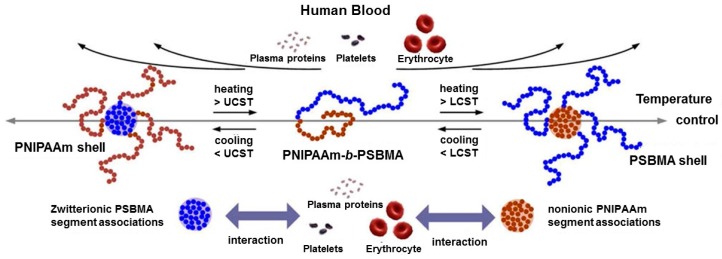
Simplified model of temperature dependence of polymer conformation in aqueous solution for P(NIPAAm)-*b*-poly(SBMA). Reproduced with permission from Reference [70], Copyright 2012, American Chemical Society.

Betaine-based poly(SBMA) has been reported to show a fibrinogen adsorption constraint that is equally matched with PEG [118,119]. Chang et al. prepared the statistical copolymers of poly(SBMA-*co*-NIPAAm)s as a blood compatible material [82]. The poly(SBMA-*co*-NIPAAm)s were prepared with different SBMA contents (100, 45.3, 29.0, 15.0, and 0 mol %), and only the 29.0 mol % copolymer showed both UCST (15 °C) and LCST (41 °C) behavior. These statistical copolymers were coated on substrates and the fibrinogen adsorption was investigated using surface plasmon resonance (SPR). As required, for application of blood compatible materials, the fibrinogen adsorption on the surface has to be less than 10 ng/cm^2^. All statistical poly(SBMA-*co*-NIPAAm)s showed around 5 ng/cm^2^ fibrinogen adsorption at 37 °C. Moreover, the statistical copolymers were exposed to the agglutination of blood test at 23 and 37 °C. Poly(SBMA-*co*-NIPAAm)-29.0 mol %, particularly, showed high anti-coagulation activity in human blood plasma. These polymeric coating materials with excellent blood compatibility can be used in blood contacting applications. Shih et al. investigated blood compatibility using PNIPAAm-*b*-poly(SBMA) block copolymers (PNIPAAm segment: LCST and poly(SBMA) segment: USCT), not statistical copolymers (Figure 10) [70]. The UCST and LCST ranges were controlled at 17–23 and 34–38 °C, respectively by controlling the chain length of each segment. These block copolymers were incubated with human fibrinogen solution (1.0 mg/mL), and the aggregation was measured at 4, 25, 37, and 40 °C. As control, PEG (4 kDa) showed aggregation (diameter >300 nm) resulting in protein adsorption even at 4 °C. Small aggregates that were less than 100 nm were observed in all PNIPAAm-*b*-poly(SBMA) block copolymers at the temperature range. Moreover, blood compatibility was investigated at the temperature range using red blood cell (RBC) hemolysis assay. NIPAAm homopolymer, particularly, 17%–25% hemolysis was observed at 37 and 40 °C which was over the LCST. Less than 2% hemolysis was observed in PNIPAAm-*b*-P(SBMA), which was equivalent to that with PEG. The well-hydrated PNIPAAm-*b*-poly(SBMA) achieved high blood biocompatibility and this block copolymer could reversibly change its structure i.e., micelle-to-unimer-to-micelle to reduce efficiently protein adsorption, membrane disruption, and platelet adhesion/activation. Dai et al. prepared the UCST-LCST ABA type triblock copolymers of poly(*N*-(3-(methacryloylamino)propyl)-*N*,*N*-dimethyl-*N*-(3-sulfopropyl) ammonium hydroxide (MPDSAH))-*b*-poly(MEO_2_MA)-*b*-poly(MPDSAH), and the block copolymers were used as DNA carrier (poly(MEO_2_MA) segment: LCST, and poly(MPDSAA) segment: UCST) [71]. The UCST tended to increase with increasing polymer concentration compared to that of the LCST. The tri-block copolymers were conjugated with deoxyribonucleic acid (DNA) due to the charged poly(MPDSAA) segment. Charged polymer-nucleic acid conjugated materials have been exploited for gene therapy application due to their high blood stability and programmed gene release [120,121]. Nanoparticles (diameter: around 81 nm) were observed by atomic force microscope (AFM) when the poly(MPDSAA)-*b*-poly(MEO_2_MA)-*b*-poly(MPDSAA) and plasmid DNA were mixed at 1/1 weight ratio. The diameter was controlled by the mixture ratios, e.g., 72 nm at low DNA content of 10/1 weight. The temperature responsive property of the conjugated tri-block copolymers/DNA (calf thymus DNA) was also investigated using transmittance change. After conjugation with DNA, the LCST was slightly increased. Interestingly, the UCST disappeared even with low DNA content (10/1 *w*/*w*). The phenomena were explained by low or no polymer aggregations arising from electrostatic repulsion between the DNA and betaine residues. 

**Figure 11:**
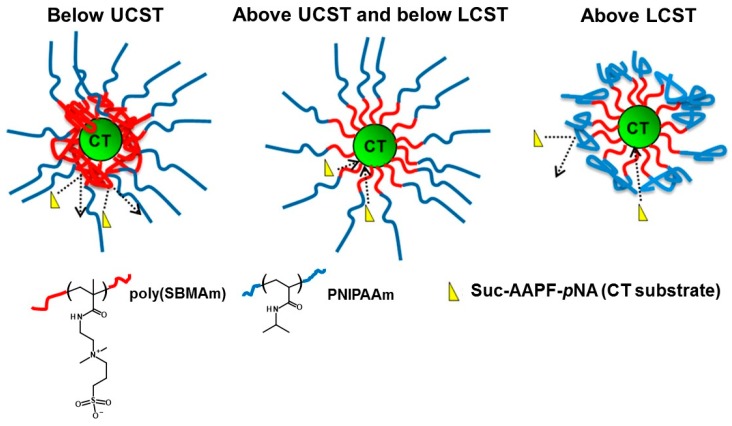
Effect of poly(SBMAm) and PNIPAAm collapses on substrate affinity. Reproduced with permission from Reference [72], Copyright 2014, American Chemical Society.

Hydrophilic polymers including PEG are covalently combined with biological macromolecules such as proteins and nucleic acids to reduce immunogenicity and prolonged circulation time [122,123]. Cummings et al. controlled the activity of protein chymotrypsin (CT) using an UCST-LCST poly(sulfobetainmethacrylamide (SBMAm))-*b*-PNIPAAm (poly(SBMAm) segment: UCST, PNIPAAm segment: LCST). The block copolymers were polymerized from CT by surface initiated atom transfer radical polymerization [72]. The CT is a serine protease that acts in the small intestine and its stability was much improved in harsh environments (temperature, low pH, and protease degradation) by polymer modification (Figure 11). The P(SBMAm)-*b*-PNIPAAm on CT could control the access of the CT-substrate due to the structures being dependent on the temperature. Moreover, the activity of modified CT was kept around 70% after incubation for 75 min at pH 1 with pepsin (bare CT completely lost its activity at the same condition). These high stabilities on modified CT were explained by hydration/dehydration of P(SBMAm)-*b*-PNIPAAm resulting in access restriction of molecules and the reducing unfolding structure.

**Figure 12:**
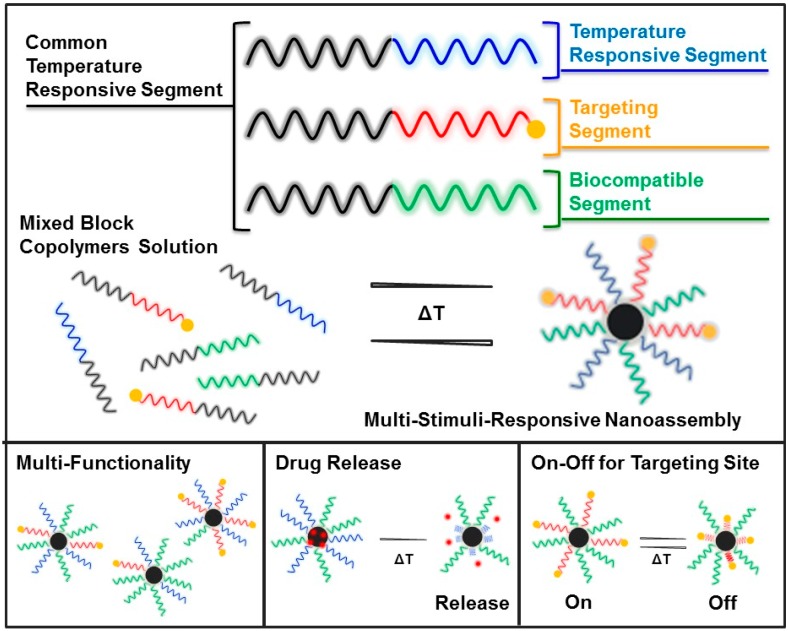
Multi-stimuli-responsive nanoassembly by mixing of selected block copolymers with a common temperature-responsive segment. Reproduced with permission from Reference [61], Copyright 2012, Royal Society of Chemistry.

Unfortunately, no in vivo studies on dual thermo-responsive block copolymers have been carried out so far. Thermo-responsive copolymers have been actively investigated in preclinical studies. For example, Li et al. prepared an immune-micelle with PNIPAAm in the shell for gastric cancer treatment [124]. The anti-Her2 antibody Fab fragments were anchored on the micelle. The immune-micelle was injected into the Balb/c nude mice bearing gastric cancer via i.v. administration and the treated tumor volume was fivetimes smaller compared with that of the control. The high stability of the micelle in blood circulation was explained by the inhibition of non-specific adsorption resulting in the high polymeric density of the shrinking PNIPAAm shell. Feng et al. applied a thermo-responsive rod-like micelle to in vivo gene therapy for nucleus pulposus regeneration [125]. The PEG and PNIPAAm were located as a rod-like micelle shell in which the shrinking PNIPAAm was used to achieve a more compact structure. The rod-like micelle loading Hemooxygenase-1 (HO-1) plasmid DNA was directly injected into the rat disc tissue space for gene therapy, and showed a high healing effect by reducing inflammatory responses and an increase in the glycosaminoglycan content. In this way, shrinking thermo-responsive polymers can increase polymeric density on nanomaterials and are expected to reduce non-specific adsorption in blood circulation. The ethylene glycol types of thermo-responsive copolymers, particularly, will be applied in this field because of their high blood compatibility. The formation of thermo-responsive copolymers in blood circulation is still simple, i.e., swelling and shrinking. The dual (or multiple) thermo-responsive nature of polymeric systems is an excellent property that can be exploited for biomedical applications. We have focused on the design of multi-stimuli responsive nano-assemblies using dual temperature responsive block copolymers, especially LCST-LCST type block copolymers. Multi-functional nano-assemblies consisting of a mixture of block copolymers having a common segment have been investigated using physical simulation [126,127]. Micelle preparation is very rapid and no purification is needed. Designed two or three shell functionalities are applied in controlled drug release and switchable on-off exposure of the targeted site (Figure 12) [37,61]. Mixed core nanoparticles were also prepared using a mixture of the self-assemble statistical and block LCST-LCST copolymers [62]. Temperature responsive statistical copolymers were effectively encapsulated in the micelle cores with high loading capacity. Moreover, a chimeric core was successfully constructed by block copolymers and specific statistical copolymers. These systems will be applied in the simple preparation of multi-stimuli responsive nano-assemblies.

## 5. Conclusions

This review provided an overview on current dual thermo-responsive block copolymers and their potential as biomedical applications. Dual thermo-responsive block copolymers can change their structural conformations as a function of the solution temperature without any additional chemicals. The block copolymers were mainly categorized via LCST-LCST and UCST-LCST types. Interestingly, there is no report on the UCST-UCST type of copolymers. The copolymers have a potential to be a micelle platform as injectable gels via a heating/cooling process. Dual thermo-responsive block copolymers can represent three property changes by each temperature sensitive segment i.e., hydrophilicity-hydrophilicity, amphiphilicity, and hydrophobicity-hydrophobicity below the critical micelle concentration (CMC). These block copolymers can be comparatively prepared using a combination of living radical polymerizations and click chemistry. The linear polymer structures have been controlled to monomer unit scale using advanced strategies [128,129,130]. For example, the glycopolymers consisting of 12–27 units of sugar residues were polymerized in multi-step polymerization for inhibition between a lectin and gp120 protein on human immunodeficiency virus (HIV) [131]. Moreover, block copolymers having over 10 segments were prepared with desirable properties in the segments [132]. In future, using these methods, multi-thermo-responsive block copolymers can be prepared. Up to now, the maximum number of thermo-responsiveness in a block copolymer is three. Even the triple thermo-responsive block copolymers can show 12 conformations depending on the solution temperature via the bonding orders of the blocks [31]. The conformation types are exponentially increased on increasing the numbers of thermo-responsive blocks. These different conformations may be applied in model proteins, encryptions, and sensors. Dual thermo-responsive copolymers have been applied in biomaterials such as nanoparticles, coating materials, and conjugated materials. The on-off switchable hydration/dehydration properties achieve controlled protein activity. Moreover, more complex and programmable orders can be added to a nanoparticle via combinations with other stimuli responses such as pH, light, and target molecules. We are convinced that these advanced dual (or multi) temperature responsive block copolymers will contribute to an important breakthrough for biomedical applications.

## Figures and Tables

**Table 1:** List of dual thermoresponsive copolymers. Numbers and alphabets refer to the polymer structures in Figure 2 and Figure 3.

**LCST-LCST type block copolymers in aqueous solution**	**Ref.**
Poly(15)-*b*-Poly(16)	[26]
Poly(16)-*b*-Poly(14)	[26,27]
Poly(15)-*b*-Poly(14)	[26,28,29]
Poly(16)-*b*-Poly(14)-*b*-Poly(15)	[30]
Poly(2)-*b*-Poly(18)-*b*-Poly(3)	[31]
Poly(2)-*b*-Poly(3)-*b*-Poly(18)	[31]
Poly(3)-*b*-Poly(2)-*b*-Poly(18)	[31]
Poly(3)-*b*-Poly(1)-*b*-Poly(4)	[32,33,34]
Poly(3)-*b*-Poly(1)	[32,34]
Poly(1)-*b*-Poly(10)	[35,36,37]
Poly(1)-*b*-Poly(6)-*b*-Poly(DMAAm)	[38]
Poly(1)-*b*-Poly(DMAAm)-*b*-Poly(6)	[38]
Poly(DMAAm)-*b*-Poly(1)-*b*-Poly(6)	[38]
Poly(3)-*b*-Poly(2)	[39]
PEG-*b*-Poly(1)-*b*-Poly(12)	[40]
Poly(23)-*b*-Poly(19)	[41]
Poly(25)-*b*-Poly(26)-*b*-Poly(25)	[42]
Poly(1)-*b*-Poly(6)	[43]
Poly(21)-*b*-Poly(21)	[44]
Poly(28)-*b*-Poly(1)	[45]
Poly(35)-*b*-Poly(33)-*b*-Poly(35)	[46]
Poly(1)-*b*-Poly(33)-*b*-Poly(1)	[47]
Poly(13)-*b*-Poly(17)	[48]
Poly(33)-*b*-Poly(MPC)-*b*-Poly(1)	[49]
Poly(1)-*b*-Poly(34)-*b*-Poly(1)	[50]
Poly(1)-*b*-Poly(11)	[51]
Poly(29)-*b*-Poly(27)	[52]
Poly(30)-*b*-Poly(31)	[53]
Poly(30)-*b*-Poly(32)	[54]
Poly(30)-*b*-Poly(32)-*b*-Poly(30)	[54]
Poly(1)-*b*-Poly(14)	[55]
Poly(14)-*b*-Poly(1)-*b*-Poly(14)	[56]
Poly(St)-*branch*-poly(1)-poly(23)	[57]
Poly(1)-*b*-Poly(23)	[58]
Poly(7)-*b*-Poly(8)	[59]
Poly(20)-*b*-Poly(9)	[60]
Poly(10)-*b*-Poly(11)	[61]
Poly(21)-*b*-Poly(22)	[62]
**LCST-UCST type block copolymers in aqueous solution**	**Ref.**
Poly(1)-*b*-Poly(A)	[63,64]
Poly(24)-*b*-Poly(C)	[65]
Poly(5)-*b*-Poly(C)	[66]
Poly(7)-*b*-Poly(D)	[59]
Poly(21)-*b*-Poly(E)	[67]
Poly(23)-*b*-Poly(F)	[68]
PEG-*b*-Poly(F)	[69]
Poly(1)-*b*-Poly(C)	[70]
Poly(A)-*b*-Poly(19)-*b*-Poly(A)	[71]
Poly(B)-*b*-Poly(1)	[72]
**Other types of LCST-UCST type copolymers**	**Ref.**
Poly(G): in aqueous solution	[73]
Poly(H): in aqueous solution	[74]
Poly(I): in alcohol	[75]
Poly(J): in mixture solution of water/alcohol	[76]
Poly(K): in mixture solution of water/alcohol	[77]
Poly(M): in ionic liquid	[78]
Poly(L): in ionic liquid	[79]
Poly(P): in dichloroethane	[80]
Poly(O): in aqueous solution	[81]
Poly(N): in aqueous solution	[82]

DMAAm: *N*,*N*-dimethylacrylamide; PEG: poly(ethylene glycol); MPC: 2-methacryloyloxyethyl phosphorylcholine; St: styrene; LCST: lower critical solution temperature; UCST: upper critical solution temperature.
